# Isolation and functional characterization of cold-regulated promoters, by digitally identifying peach fruit cold-induced genes from a large EST dataset

**DOI:** 10.1186/1471-2229-9-121

**Published:** 2009-09-22

**Authors:** Andrés Tittarelli, Margarita Santiago, Andrea Morales, Lee A Meisel, Herman Silva

**Affiliations:** 1 Millennium Nucleus in Plant Cell Biotechnology (MN-PCB), Santiago, Chile; 2 Plant Functional Genomics & Bioinformatics Lab, Universidad Andrés Bello, Santiago, Chile; 3 Centro de Biotecnología Vegetal, Universidad Andrés Bello, Santiago, Chile

## Abstract

**Background:**

Cold acclimation is the process by which plants adapt to the low, non freezing temperatures that naturally occur during late autumn or early winter. This process enables the plants to resist the freezing temperatures of winter. Temperatures similar to those associated with cold acclimation are also used by the fruit industry to delay fruit ripening in peaches. However, peaches that are subjected to long periods of cold storage may develop chilling injury symptoms (woolliness and internal breakdown). In order to better understand the relationship between cold acclimation and chilling injury in peaches, we isolated and functionally characterized cold-regulated promoters from cold-inducible genes identified by digitally analyzing a large EST dataset.

**Results:**

Digital expression analyses of EST datasets, revealed 164 cold-induced peach genes, several of which show similarities to genes associated with cold acclimation and cold stress responses. The promoters of three of these cold-inducible genes (*Ppbec1*, *Ppxero2 *and *Pptha1*) were fused to the GUS reporter gene and characterized for cold-inducibility using both transient transformation assays in peach fruits (*in fruta*) and stable transformation in *Arabidopsis thaliana*. These assays demonstrate that the promoter *Pptha1 *is not cold-inducible, whereas the *Ppbec1 and Ppxero2 *promoter constructs are cold-inducible.

**Conclusion:**

This work demonstrates that during cold storage, peach fruits differentially express genes that are associated with cold acclimation. Functional characterization of these promoters in transient transformation assays *in fruta *as well as stable transformation in Arabidopsis, demonstrate that the isolated *Ppbec1 *and *Ppxero2 *promoters are cold-inducible promoters, whereas the isolated *Pptha1 *promoter is not cold-inducible. Additionally, the cold-inducible activity of the *Ppbec1 *and *Ppxero2 *promoters suggest that there is a conserved heterologous cold-inducible regulation of these promoters in peach and Arabidopsis. These results reveal that digital expression analyses may be used in non-model species to identify candidate genes whose promoters are differentially expressed in response to exogenous stimuli.

## Background

Cold temperature is an environmental factor that plays an important role in plant growth and development. Temperate plants have developed mechanisms to adapt to periods of low non-freezing temperatures, enabling these plants to survive subsequent freezing temperatures. This process is called cold acclimation [1]. Cold acclimation is a complex process that involves physiological, biochemical and molecular modifications [2-4]. Hundreds of genes have been shown to have altered expression levels during cold acclimation [5]. These alterations enable the plant to withstand freezing by creating a chronic response that protects the integrity of the cellular membranes, enhances anti-oxidative mechanisms and accumulates molecular cryoprotectants [6].

Under normal conditions, cold acclimation is initiated by the cold temperatures of late fall and early winter, when fruit trees lack fruits. Similar cold temperatures have been used in the fruit industry to store fruits for prolonged periods of time. These temperatures inhibit fruit ripening, thereby extending fruit postharvest life. Despite the benefits, peaches that are subjected to long periods of cold storage can develop chilling injury symptoms (i.e. woolliness and internal breakdown) which reduce the postharvest quality of these fruits and results in significant economical losses [7-9].

Most of the efforts directed towards understanding the molecular basis of cold acclimation have been performed in the model plant *A. thaliana *[1-4]. Little is known about what occurs under low, non-freezing temperatures in fruits or fruit trees. Since chilling injury occurs in fruits that have undergone long-term cold storage, perhaps cold acclimation processes are associated with this injury. A better understanding of cold acclimation and cold-responsive genes in peach trees and fruits may provide clues about the association of cold acclimation and chilling injury.

Several transcription factors associated with cold acclimation have been shown to regulate the expression of cold-inducible genes containing conserved ABRE (abscisic acid response elements) and/or DRE (dehydration-responsive) elements in their promoters [10-13]. The regulation of cold-inducible promoters in peaches may be mediated by the interaction between promoters containing these types of cis-elements and orthologous transcription factors. However, the identification and functional characterization of these types of promoters in fruit trees is lacking.

We have demonstrated previously that there is a conserved heterologous regulation of the wheat putative high-affinity Pi transporter, *TaPT2 *in both monocots (wheat) and dicots (Arabidopsis) [14]. These findings demonstrate that Arabidopsis may be used as a heterologous system to test the functionality of promoters. However, this type of heterologous regulation may not exist for all promoters and may not be conserved among all plant species. An alternative to functional analyses in heterologous systems is transient transformation of fruits using agro-infiltration. Agro-infiltration of fruits have been performed to test the activity of the 35S CaMV promoter fused to reporter genes such as GUS or luciferase in tomatoes, apples, pears, peaches, strawberries and oranges [15,16]. However, to our knowledge, it has not been used to determine the activity of cold-inducible promoters within the fruit (*in fruta*).

To identify cold-responsive genes expressed in peach fruits, digital expression analyses of ESTs from fruits exposed to four different postharvest conditions were analyzed [17]. Isolation of the promoter regions of three genes highly expressed in fruits that have undergone long-term cold storage, allowed us to identify common regulatory elements present in these promoters. Functional characterization of these promoters (stably in *A. thaliana *and transiently in peach fruits) demonstrates that these are peach cold-inducible promoters and that there is a conserved heterologous regulation of these promoters in peach and Arabidopsis.

## Methods

### Digital expression analyses

We have previously described the contigs used in this work [17]. The ESTs that make up these contigs represent transcripts from peach fruit mesocarp at four different postharvest conditions. The post-harvest conditions include: fruits processed in a packing plant (E1: non-ripe; no long term cold storage); packing followed by a shelf-life at 20°C for 2-6 days (E2: Ripe; no long term cold storage; juicy fruits); packing followed by cold storage at 4°C for 21 days (E3: non-ripe; long term cold storage) and packing followed by cold storage at 4°C for 21 days and shelf-life at 20°C for 2-6 days (E4: Ripe; long term cold storage; woolly fruits).

As we described in Vizoso et al [17], the contigs that represent differentially expressed genes were identified using the Winflat program that submits the sequence data to a rigorous statistical analysis described by Audic and Claverie [18]. This analysis calculates the probability that a gene is equally expressed in two different conditions by observing the distribution of tag counts (number of ESTs). Therefore, small probability values (*p*-values) are associated with non-symmetrical distributions, characteristic of differentially expressed genes [18,19].

To analyze the co-expression of differentially expressed genes, contigs were clustered using the Pearson linear correlation coefficient [19,20]. Briefly, contigs with at least five ESTs were selected to make the expression profile matrix, which consisted of 1,402 rows (the contigs) and 4 columns (four cDNA libraries). The similarity between clusters and libraries was estimated using an un-centered Pearson's correlation coefficient in the Cluster 3.0 program [20]. Pearson correlation coefficients > 0.85 (zero values indicate no association and a coefficient equal to 1 indicate a fully correlated pattern) are indicated by an asterisk in Additional File 1. Dendrograms were constructed from the pair wise distances using the UPGMA algorithm. The results were visualized and analyzed using the Java TreeView program .

Gene Ontology molecular function and biological process annotations of the contigs are described in Vizoso et al [17]. Each annotation and contig assembly was manually corrected, when necessary.

### mRNA isolation and reverse transcriptase (RT)-PCR

The kit Oligotex™ mRNA Spin-Column (Qiagen, New York, USA) was used to purify mRNA. The mRNA was purified from pools of total RNA obtained from peach fruit mesocarp (*O'Henry *var.) representing the stages E1, E2, E3 and E4 as described previously [17,21]. The mRNA was quantified using the Poly (A) mRNA Detection System™ (Promega, Madison, USA). First strand cDNA was synthesized from 5 ng of the mRNA in a 20 μl final volume. The reaction mix was prepared using the ImProm-II™ reverse Transcription System (Promega, Madison, USA) and anchored oligo (dT) of 18-mers, according to the manufacturer's instructions. As an internal control for normalization, heterologous mRNA (1.2 kb mRNA coding for Kanamycin) was added to each mRNA sample. To control for genomic DNA contamination, PCR amplification was performed on template RNA that was not reverse transcribed. To confirm that the amplified fragments correspond to the cDNAs of interest, these fragments were cloned in pBluescript and sequenced (Macrogen, Korea). The primer sequences used to amplify the internal regions of the basic endochitinase *Ppbec1 *(BEC226F and BEC576R), dehydrin *Ppxero2 *(DX-82F and DX176R), thaumatin *Pptha1 *(THA30F and THA382R), lipoxygenase *Pplox1 *(LOX982F and LOX1267R) and the actin *Ppact7 *(ACT-F and ACT-R) genes are shown in Table 1. Primers used to amplify a 323 bp fragment of the cDNA from the Kanamycin mRNA control are: "Upstream Control Primer" (5'-gCCATTCTCACCggATTCAgTCgTC-3') and "Downstream Control Primer" (5'-AgCCgCCgTCCCgTCAAgTCAg-3'). PCR reactions were performed by diluting the cDNAs a 100 fold and using 1 μl of each dilution as a template in a final reaction volume of 20 μl, containing 0.5 μM primers; 0.2 mM dNTPs; 1.5 mM MgCl_2_; 5U Taq polymerase and 1× buffer. The PCR conditions were: 93°C for 5 min and then a variable number of cycles (26 to 34) at 93°C for 30 sec, 1 min at 55°C, and 1 min at 72°C. The PCR reaction was with a final step at 72°C for 10 min.

**Table 1:** Primers used in this study

**Primer**	**Sequence (5'→3')**	**Method**
BEC226F	gTCAgCAgCgTCgTTAgCTC	RT-PCR
BEC576R	gAgTTggATgggTCCTCTgC	
DX-82F	CCAAACCAAAgCCAgTTTCATTCA	
DX176R	CCAggTTTTgTATgAgTgCCgTA	
THA30F	ACCTTggCCATCCTCTTCTT	
THA382R	AgAAATCTTgACCCCCgTTC	
LOX982F	AAggAgCTCTTgACgTTggA	
LOX1267R	TgCTAACAggTgggAAAACC	
ACT-F	CCTTCCAgCAgATgTggATT	
ACT-R	AgATTAggCAAggCgAggAT	

BEC87-GSP1	TgCATTTCCAgCTTgCCTCCCACATTg	Genome Walker
BEC55-GSP2	CTgAgATCCCTAACAgCAAAgCTAgggATA	
DX85-GSP1	ACCggTTCCggTggTggTgTgATgAACC	
DX46-GSP2	ACTCATCAgTCTTAgTAggCTCgggTgTT	
THA82-GSP1	TgATTTTAgCTgCATgTgCACCTgAgAA	
THA-1-GSP2	CgTCATggAAATgTCTTAATTggCTTgCTg	
LOX101-GSP1	gAAgAAAACAAATTgggAggAggAgAA	
LOX63-GSP2	gCgTgTTCCAAAgAACACAATTCAgTgCCTT	

BEC-32BamHI	ggATCCTgATCTgTggATTgggTTTCgTgg	Subcloning promoters
DX24BamHI	ggATCCgggTgTTgAACCAAAATgCgCCATT	

### Cloning of the promoters

Genomic DNA was isolated from peach leaves (*Prunus persica *var. persica (L.) Batch cv. *O'Henry*) as described in Manubens et al [22]. The Universal Genome Walker™ Kit (Clontech Laboratories, Inc., Palo Alto, CA, USA) was used to isolate the promoters regions of *Ppbec1*, *Ppxero2*, *Pptha1 *and *Pplox1*. The isolated genomic DNA was digested with four restriction enzymes (*Eco*RV, *Pvu*II, *Ssp*I, and *Mls*I). DNA fragments containing adaptors at both ends were used as a template for amplifying the promoter regions. GSP1 and GSP2 gene specific primers were designed to isolate the promoters (Table 1). For the first group of PCR reactions, a specific adaptor primer (AP1, 5'-ggATCCTAATACgACTCACTATAgggC-3') and the GSP1 primers specific for each gene were used. The final primer concentration in the PCR reaction was 0.2 μM in a final volume of 50 μL. Manual Hot Start was performed using 5 U of the Synergy DNA polymerase (Genecraft, Münster, Germany). The conditions for this first round of amplifications was: 1 cycle at 93°C for 10 min, 7 cycles of 93°C for 30 sec, 72°C for 15 min, followed by 37 cycles of 93°C for 30 sec, 67°C for 15 min. For the nested PCR, the specific adaptor primer 2 (AP2, 5'-ACTATAgggCACgCgTggT-3') and the gene specific GSP2 primers were used. As a DNA template in these reactions, 1 μL of a 50 fold dilution of end-product of the first round of amplifications was used. The conditions for the second round of amplification were: 1 cycle at 93°C for 10 min, 5 cycles (7 cycles in the case of *Ppxero2*) of 93°C for 30 sec, 72°C for 15 min, followed by 20 cycles (30 cycles in the case of *Ppxero2*) of 93°C for 30 sec, 67°C for 15 min. The amplified products were cloned in pGEM-T vector and sequenced (Macrogen, Korea). The *Ppbec1 *and *Ppxero2 *promoters were subsequently amplified from the pGEM-T clones using the AP2 and BEC-32BamHI or DX24BamHI primers, respectively (Table 1). The products of this amplification were also cloned in the pGEM-T vector and re-sequenced (Macrogen, Korea). The promoter fragments were extracted from the pGEM-T vector (including the *Pptha1 *promoter), with a *Bam*HI-*Sal*I sequential digestion, and transcriptionally fused to the *uidA *reporter gene in the promoterless binary vector pBI101.1 [23]. The binary vector was introduced into *A. tumefaciens *(GV3101) for subsequent Arabidopsis and peach fruit transformations.

### Promoter sequences analysis

Analysis of putative transcription factor binding sites was carried out using the database PLACE [24] coupled with visual analyses. To identify predicted conserved motifs, the promoter sequences were analyzed using the YMF 3.0 program [25]. Only the statistically significant motifs (Z score value > 6.5) were selected [26].

### Growth, transformation and cold treatments of A. thaliana

Wild-type and transgenic *A. thaliana *(ecotype Columbia) were grown in a mixture of soil-vermiculite (3:1) in a growth chamber with a 16-h light cycle (140 μmol m^-2 ^s^-1^) at 22°C. Alternatively, seeds were surface sterilized as described in Gonzalez et al [27], plated on Murashige-Skoog (1 × MS) media containing 0.8% agar, 0.1% sucrose and 50 mg/l Kanamycin for transgenic lines and grown under the same conditions as the soil-grown plants.

Transgenic Arabidopsis was obtained by using the GV3101 *A. tumefaciens*-mediated floral dip method [28]. *A. tumefaciens *previously transformed with the binary vector pBI101.3 harboring the promoter::*uidA *fusions: *Ppbec1*::*uidA *(PBIPpbec1); *Ppxero2*::*uidA *(pBIPpxero2); *Pptha1*::*uidA *(pBIPptha1), or the control vectors pBI121 (containing the 35S CaMV promoter) and pBI101.3 (promoterless), were used. In cold treatments, T_3 _homozygous transgenic Arabidopsis seedlings were grown on plates containing 1× MS media, 0.8% agar, and 0.1% sucrose in a growth chamber with a 16-h light cycle (140 μmol m^-2 ^s^-1^) at 24°C for two weeks, and then transferred to 4°C for 7 days. A minimum of three independent transgenic lines were used for each construct.

### Peach fruit transient transformation and cold treatments

*A. tumefaciens *transformed with the vectors pBIPpbec1, pBIPpxero2, pBIPptha1, pBI121 or pBI101.3 were grown in LB medium supplemented with Kanamycin (100 μg/ml), Rifampicin (10 μg/ml) and Gentamycin (100 μg/ml). The cultures were grown for two days at 28°C until they reached an OD_600 _between 0.6 and 0.8. The culture was then centrifuged and the pellet re-suspended in MMA medium (1× MS, MES 10 mM (pH 5.6), 20 g/l sucrose, and 200 μM acetosyringone) to reach an OD_600 _of 2.4. Approximately 0.7 mL of this bacterial suspension was used to infiltrate mature fruits from *O'Henry*, *Elegant Lady *and *Florida King *varieties of peach as described by Spolaore et al [15].

To analyze the promoter activity at 20°C, the fruits infiltrated with the different constructs, were stored in a dark growth chamber for five days. To analyze the cold-responsive promoter activity, the infiltrated fruits were stored 2 days post-infiltration (dpi) in a dark growth chamber at 4°C for 10 days. After the growth chamber incubation time, the infiltrated region of the fruit was extracted with a cork bore and stained for GUS activity as described by Tittarelli et al [14].

### GUS activity measurement

Histochemical staining of Arabidopsis seedlings for β-glucuronidase (GUS) activity was performed as described by Jefferson et al [23], with the following modifications: transgenic Arabidopsis seedlings used in the cold-treatments described earlier were vacuum infiltrated in 50 mM NaH_2_PO_4_, pH 7.0; 0.1 mM X-Gluc; 10 mM EDTA and 0.1% Triton X-100. These samples were incubated in the dark at 37°C for 24-72 h. Samples that did not develop color after 72 h were considered negative for GUS activity. Plant material was subsequently fixed in 0.04% formaldehyde, 0.04% acetic acid and 0.285% ethanol for 30 min, followed by an ethanol dilution series to remove chlorophyll from the plant tissue (70% ethanol for 1 h, 100% ethanol for 1 h, 70% ethanol for 1 h and distilled water).

Slices (2 mm) of transiently transformed peaches were imbibed in the GUS staining solution (0.72 M K_2_HPO_4_; 0.17 M KH_2_PO_4_; 0.5 mM K_3_Fe(CN)_6_; 0.5 mM K_4_Fe(CN)_6_; 1× Triton X-100; 12.7 mM EDTA; 20% (v/v) methanol and 0.5 mM X-Gluc) [15]. Samples were vacuum-infiltrated for 30 min at room-temperature and then incubated overnight at 37°C. Fluorometric GUS assays were performed as described by Jefferson et al [23]. The Arabidopsis seedlings were ground in a mortar using liquid nitrogen, and the tissue powder was transferred to a microtube. One ml of the extraction buffer (50 mM NaH_2_PO_4_, pH 7.0; 1 mM EDTA; 0.1% Triton X-100; 0.1% (w/v) sodium laurylsarcosine and 5 mM dithiothreitol) was added. Samples were centrifuged for 10 min at 12,000 g at 4°C and the supernatant was transferred to a new microtube. The fluorogenic reaction was carried out in 2 ml volume containing 1 mM 4-methyl umbelliferyl glucuronide (MUG) in an extraction buffer supplemented with a 50 μL aliquot of the protein extract supernatants. The protein quantity of the sample extracts was determined as described previously [29], using bovine serum albumin (BSA) as a standard.

## Results

### Identification of peach cold-regulated genes by digital expression analyses of EST datasets

Coordinated gene expression analyses of peach fruit ESTs datasets revealed 10 major hierarchical clusters (Additional File 1), containing unique contigs. We identified 164 contigs with preferential expression in fruits stored at 4°C (E3: non-ripe; long term cold storage). Table 2 contains a complete list of these contigs together with their annotations, GO biological process annotations and the origin of the ESTs in each contig. Contigs with statistically differential expression, in E3 compared to the other stages are also indicated.

**Table 2:** Putative function of 164 genes preferentially expressed in cold stored peach fruits.

**Contig**	**E3**	**E1+E2+E4**	**AC test^1^**	**Putative Function;Arabidopsis ortholog^2^**
***Biological process unknown (GO:0000004)***				
C517	10	6	E4	NC domain-containing protein (located in mitochondrion); At5g06370
C675	12	4	E2; E4	Expressed protein; **At3g03870↓**
C774*	11	4	E2; E4	Novel gene
C2089	20	0	E1; E2; E4	Expressed protein (located in endomembrane system); At5g64820
C2112	31	2	E1; E2; E4	Cupin family protein (nutrient reservoir activity); At1g07750
C2139	12	0	E1; E2; E4	Novel gene
C4065	13	8	E2	Expressed protein; **At5g52870↑**
C273	5	2		Expressed protein; **At5g24660↓**
C477	7	6		Expressed protein (located in endomembrane system); **At5g64510↓**
C1207*	8	7		Novel gene
C2134	3	2		Expressed protein; At1g71080
C2148	4	1		Novel gene
C2155	4	1		Expressed protein; At5g11730
C2167	3	2		RWD domain-containing protein; At1g51730
C2173	7	1		Expressed protein (located in mitochondrion);**At5g60680↑**
C2193	3	2		Novel gene
C2211	8	1		Ankyrin repeat family protein (protein binding); At2g28840
C2241	6	2		Expressed protein (located in mitochondrion); At5g51040
C2267	7	0		Integral membrane family protein; At4g15610
C2315	5	3		Expressed protein; At1g70780
C2318	3	2		Ribosome associated membrane protein RAMP4; At1g27350
C2343	9	9		Novel gene
C2560	6	1		Expressed protein; **At3g27880↑**
C2591	6	1		Expressed protein (located in mitochondrion); At5g24600
C2682*	4	2		N-methyl-D-aspartate receptor-associated protein; At4g15470
C2713	4	1		Glycine-rich protein; At4g22740
C2778	12	7		Zinc finger (AN1-like) family (DNA and zinc ion binding); **At3g52800↑**
C2806	8	2		C2 domain-containing protein; At1g22610
C3094	3	2		Reticulon family protein (located in ER and mitochondrion); **At3g10260↓**
***Cell homeostasis (GO:0019725)***				
C2265	91	38	E1; E2; E4	Metallothionein-like protein; At5g02380
C2202*	5	1		Metallothionein-like protein; NSM^4^
***Cell organization and biogenesis (GO:0016043)***				
C734	17	9	E2; E4	Proline-rich/extensin family; At2g27380
C1240	62	20	E1; E2; E4	Proline-rich/extensin family; At1g54215
C2494*	10	3	E2	Actin-depolymerizing factor 4; At5g59890
C2831	20	6	E1; E2; E4	Leucine-rich repeat/extensin family; **At4g13340↑**
C3041	12	5	E2; E4	Leucine-rich repeat/extensin family; **At4g13340↑**
C831	4	2		BON1-associated protein (BAP2); **At2g45760↑**
C1062	4	1		Invertase/pectin methylesterase inhibitor family; **At5g62360↑**
C2060	7	3		Expansin family; **At4g38400↑**
C2086*	6	1		Arabinogalactan-protein; At5g64310
C2073	6	2		Zinc finger protein (CYO1); At3g19220
C2574	7	3		Invertase/pectin methylesterase inhibitor family; At2g01610
C2762*	4	1		Profilin 4; At2g19770
C2815	4	1		Phytochelatin synthetase; At4g16120
***Cellular protein metabolism (GO:0044267)***				
C228*	112	51	E1; E2; E4	DJ-1 family protein/protease-related; **At3g02720↓**
C379*	50	21	E1; E2; E4	DJ-1 family protein/protease-related; **At3g02720↓**
C1027*	47	46	E1; E2; E4	Heat shock cognate 70 kDa protein 1; At5g02500
C1660	51	25	E1; E2; E4	Cysteine proteinase inhibitor-related; **At2g31980↓**
C2099*	13	1	E1; E2; E4	DJ-1 family protein/protease-related; **At3g02720↓**
C2436	17	3	E1; E2; E4	Rhomboid family protein; At1g63120
C2715	41	21	E1; E2; E4	Luminal binding protein 1 (BiP-1); At5g28540
C2066*	3	2		60S ribosomal protein L23A; **At3g55280↑**
***Cellular protein metabolism (GO:0044267)***				
C2072*	6	2		DNAJ heat shock protein; At3g44110
C2217*	7	3		20S proteasome beta subunit A; At4g31300
C2308*	9	0		Heat shock protein 70; **At3g12580↓**
C2345*	4	2		Ubiquitin carrier protein E2; At2g02760
C2364	5	2		Phosphatase-related (SGT1B); At4g11260
C2388	5	3		F-box family protein (AtSKP2;2); At1g77000
C2593	4	1		C3HC4-type RING finger family protein; **At1g26800↓**
C2597	6	2		26S proteasome regulatory subunit S3; At1g20200
C2691	7	6		C3HC4-type RING finger family protein; At5g47610
C2360	10	7		Structural constituent of ribosome; At5g15260
C2735	9	4		40S ribosomal protein S9; At5g39850
C3022	6	2		Translation initiation factor IF5; At1g36730
C3051*	5	2		DJ-1 family protein/protease-related; **At3g02720↓**
C3520	4	1		60S ribosomal protein L36; At3g53740
C3551*	11	4		Cysteine proteinase inhibitor; **At3g12490↑**
C3656	6	4		40S ribosomal protein S26; At3g56340
C4131	3	2		C3HC4-type RING finger family protein; At5g48655
***Development (GO:0007275)***				
C2802	10	2	E1	Senescence-associated protein; **At1g78020↓**
C2919	10	1	E1; E2	Senescence-associated protein; At5g20700
C1113	6	3		Auxin-responsive protein; **At3g25290↓**
C3887*	4	1		Maternal effect embryo arrest 60; At5g05950
C3942	6	4		SIAMESE, cyclin binding protein; **At5g04470↓**
C2457	6	0		Nodulin MtN3 family protein; **At5g13170↑**
***Generation of precursor metabolites and energy (GO:0006091)***				
C2304	7	1		NADH dehydrogenase; **At4g05020↑**
C2541	8	1		Uclacyanin I; **At2g32300↓**
C2552	5	0		Flavin-containing monooxygenase family protein; **At1g48910↑**
***Metabolism (GO:0008152)***^3^				
C1017	15	9	E2	Xyloglucan endotransglycosylase; **At4g25810↓ ***(carbohydrate)*
C1258*	19	2	E1; E2; E4	Phosphoesterase family protein; **At3g03520↓ ***(phospholipid)*
C2373	15	8	E2; E4	β-alanine-pyruvate aminotransferase; **At2g38400↓ ***(amino acid)*
C2397*	27	9	E1; E2; E4	S-adenosylmethionine decarboxylase; At3g02470 *(polyamine)*
C2554*	17	3	E1; E2; E4	UDP-glucoronosyl/UDP-glucosyl transferase; At5g65550 *(anthocyanin)*
C2957	11	0	E1; E2; E4	Glycosyl hydrolase family 3; **At5g49360↓ ***(carbohydrate)*
C2669	61	28	E1; E2; E4	Phosphoserine aminotransferase; At4g35630 *(amino acid)*
C656	4	3		Nucleoside diphosphate kinase 3; At4g11010 *(nucleotide)*
C821*	4	1		UDP-glucoronosyl/UDP-glucosyl transferase; **At5g49690↑ ***(anthocyanin)*
C926*	7	6		(1-4)-β-mannan endohydrolase; **At5g66460↑ ***(carbohydrate)*
C1000*	8	2		Alkaline alpha galactosidase; At1g55740 *(carbohydrate)*
C1693	9	3		Haloacid dehalogenase-like hydrolase; **At5g02230↓**
C1943	4	3		2-oxoglutarate-dependent dioxygenase; At1g06620 *(ethylene)*
C2424	5	0		β-amylase; **At4g17090↑ ***(starch)*
C2495	8	1		Cinnamoyl-CoA reductase; At4g30470 *(lignin)*
C2522	11	8		Glycosyl hydrolase family 5; **At1g13130↑ ***(carbohydrate)*
C2569	7	1		Short-chain dehydrogenase/reductase family; **At3g61220↓**
C2602	5	0		Short-chain dehydrogenase/reductase family; At4g13250
C2610	5	0		Galactinol synthase; **At3g28340↑ ***(carbohydrate)*
C2222	6	0		Carboxyesterase 5; **At1g49660↓**
C2635	6	4		GNS1/SUR4 membrane family protein; At4g36830 *(fatty acid)*
C2705	7	4		DSBA oxidoreductase family protein; At5g38900 *(organic acid)*
C669	4	2		Dehydrogenase; At5g10730
C2936	4	1		Pyruvate decarboxylase; At5g17380 *(glycolisis)*
C2940	4	1		Farnesyl pyrophosphate synthetase 1; At5g47770 *(lipid)*
C2976	6	1		Aminoalcoholphosphotransferase; At1g13560 *(phospholipid)*
C3047*	7	4		Dienelactone hydrolase; At3g23600 *(alkene)*
***Metabolism (GO:0008152*)**^3^				
C3058*	5	1		Cellulose synthase; At4g39350 *(cellulose)*
C3152	8	3		Purple acid phosphatase; At3g52820 *(phosphate)*
C3225	4	1		Acyl-activating enzyme 12; At1g65890 *(phospholipid)*
C4127	6	2		Ω-3fatty acid desaturase; At5g05580 *(fatty acid)*
C86	6	3		Embryo-abundant protein; **At2g41380↑**
C677	4	2		Cyclic phosphodiesterase; At4g18930 *(RNA)*
C802	4	3		RNA recognition motif-containing protein; At5g04600 *(RNA)*
C2798	3	2		Small nuclear ribonucleoprotein G; **At2g23930↑ ***(RNA)*
***Response to stress (GO:0006950)***				
C30	57	27	E1; E2; E4	Cold acclimation WCOR413-like protein; At3g50830
C254	71	10	E1; E2; E4	Dehydrin Xero2; **At3g50970↑**
C304*	189	124	E1; E2; E4	Type II dehydrin SKII; (ERD14) **At1g76180↑**
C910	126	38	E1; E2; E4	Class III acidic endochitinase; At5g24090
C1479	96	25	E1; E2; E4	Harpin inducing protein; **At5g06320↑**
C1708	30	12	E1; E2; E4	Thaumatin-like protein; **At1g20030↑**
C2131	65	2	E1; E2; E4	Class Ib basic endochitinase; **At3g12500↑**
C2177	15	4	E1; E4	Thaumatin-like protein; **At1g20030↑**
C2317	67	6	E1; E2; E4	Thaumatin-like protein; **At1g20030↑**
C2514*	20	15	E2	Glutathione peroxidase; **At4g11600↑**
C2528	22	7	E1; E2; E4	Hevein-like protein; **At3g04720↓**
C2655*	10	6	E4	DREPP plasma membrane polypeptide; At4g20260
C2988*	37	6	E1; E2; E4	Polygalacturonase inhibiting protein; **At5g06860↑**
C2473*	10	0	E1; E2; E4	Major allergen Pru p 1; At1g24020
C2147	8	0		Thaumatin-like protein; **At1g20030↑**
C2441	8	1		Class IV chitinase; At3g54420
C2507	5	2		Pyridoxine biosynthesis protein; At5g01410
C2556	5	0		4-aminobutyrate aminotransferase; At3g22200
C2578	3	2		Aldehyde dehydrogenase; At1g44170
C2926	7	2		Wounding stress inducimg protein; At4g24220
C3613*	3	2		Harpin inducing protein; At3g11660
C1889*	5	4		Major allergen Pru p 1; At1g24020
C3858*	4	2		Late embryogenesis abundant protein 3; **At4g02380↑**
***Signal transduction (GO:0007165)***				
C815	9	1		Leucine-rich repeat family protein; At3g49750
C1192*	6	5		CBL-interacting protein kinase 12; **At4g18700↑**
C2205	5	4		Ser/Thr kinase; **At2g47060↓**
C2312*	8	3		Touch-responsive/calmodulin-related protein 3; **At2g41100↓**
C2430*	6	6		Remorin family protein; **At5g23750↓**
C2548	10	6		Fringe-related protein; At4g00300
C2829*	3	2		Protein kinase, 41K; **At5g66880↓**
C2853	5	3		GTP-binding protein Rab2; At4g17170
C3690*	10	8		Ser/Thr kinase; At4g40010
***Transcription (GO:0006350)***				
C452	4	2		Myb family; At5g45420
C2742*	5	1		DREB subfamily A-6; **At1g78080↓**
C3420*	8	4		MADS-box protein (AGL9); At1g24260
C3812	3	2		WRKY family; **At4g31550↑**
***Transport (GO:0006810)***				
C716	13	5	E2; E4	Proton-dependent oligopeptide transport family; **At5g62680↓**
C1846	15	10	E4	Auxin efflux carrier family protein; **At2g17500↓**
C2091	18	0	E1; E2; E4	Protease inhibitor/seed storage/lipid transfer family; At1g62790
C163	4	1		Vesicle-associated membrane protein; At1g08820
C208	9	2		GTP-binding secretory factor SAR1A; At4g02080
C235	5	4		Sugar transporter; **At1g54730↓**
C484	11	6		Porin; At5g67500
C1526	5	4		emp24/gp25L/p24 protein; At3g22845
***Transport (GO:0006810)***				
C2062	3	2		Ripening-responsive protein; At1g47530
C2236	3	2		Ras-related GTP-binding protein; At4g35860
C2476	9	1		Bet1 gene family; **At4g14450↑**
C2679	5	0		Sulfate transporter ST1; **At3g51895↑**
C3063	4	2		Amino acid carrier; **At1g77380↑**
C3066	4	1		Sulfate transporter; At3g15990
C3099	3	2		Ras-related GTP-binding protein; At1g52280

^1 ^Statistically significant cold-induced contigs detected with the Audic and Claverie test (*p *< 0.01) *vs*. E1, E2 or E4 cDNA libraries. The column shows the cDNA library with differences to E3.

^2^The column described the locus identifier (id) of the Arabidopsis most similar protein. The locus ids with **↑ **[37] are the Arabidopsis cold response genes similarly up-regulated; the locus ids with **↓ **[31] are the genes with opposite response, down-regulated in Arabidopsis (ColdArrayDB; ).

^3^Between parentheses: the principal subcategory of the biological process "metabolism" associated to the annotation.

^4^NSM: Not significant match (E value < 10^-10^) with *A. thaliana *sequences.

* Contigs that shown significant sequence homology (e value > 10^-10^) with contigs from others hierarchical clusters.

Approximately 95% of the 164 cold-induced peach genes share significant identity with sequences in Arabidopsis, suggesting that these may be putative orthologs. The putative Arabidopsis orthologs that are induced or repressed by cold, based on ColdArrayDB analyses  are shown in Table 2. Only 29 contigs (18% of the 164 cold-induced genes) share significant sequence identity with genes of unknown function. Approximately 38% of these contigs (11 contigs) share significant sequence identity with plant gene sequences annotated as expressed proteins. Six of the contigs with unknown function do not share sequence identity with any sequences in the public databases, suggesting that these are novel genes.

**Figure 1:**
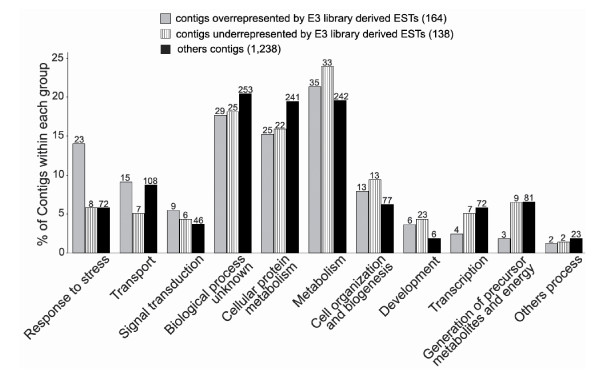
**Annotation frequency comparison of cold-induced, cold-repressed or unrelated to cold-induction contigs**. The frequency of contigs that are associated with a specific Gene Ontology are expressed as the percentage of the total annotations for each analyzed group (164 for the cold-induced, 138 for the cold-repressed and 1,238 for unrelated to cold-induction). The numbers of contigs in each group, belonging to each biological process classification, are show at the top of each bar. The category "others process" are: cell adhesion (GO: 0007155, 1 contig); cell communication (GO: 0007154, 1 contig); cell cycle (GO: 0007049, 5 contigs); cell death (GO: 0008219, 1 contig); cell homeostasis (GO: 0019725, 4 contigs); organism physiological process (GO: 0050874; 1 contig); regulation of GTPase activity (GO: 0043087; 1 contig); response to stimulus (GO: 0050896; 10 contigs) and viral life cycle (GO: 0016032; 1 contig).

Annotation frequency comparative analyses of cold-induced (164 contigs), cold-repressed (138 contigs) or contigs unrelated to cold (1,238 contigs), revealed an overrepresentation of stress response genes and an underrepresentation of genes related to energy metabolism in fruits that were stored in the cold (Figure 1). Among the genes related to stress response we identified four contigs that are similar to thaumatin-like proteins: C1708, C2177, C2317 and C2147 (98%, 99%, 98% and 93% amino acid identity with *P. persica *thaumatin-like protein 1 precursor, respectively, GenBank accession number: P83332). Three of the stress response genes are similar to chitinases: C910 (76% amino acid identity with *Malus domestica *class III acidic endochitinase, GenBank accession number: ABC47924); C2131 (74% amino acid identity with *Galega orientalis *class Ib basic endochitinase, GenBank accession number: AAP03087) and C2441 (72% amino acid identity with *A. thaliana *class IV chitinase, GenBank accession number: NP_191010). Two of the stress response genes are similar to dehydrins: C254 (97% amino acid identity with *P. persica Ppdhn1*, GenBank accession number: AAC49658) and C304, 100% amino acid identity with *P. persica *type II SK2 dehydrin *Ppdhn3 *(Genbank accession number: AAZ83586).

### Cold-induced expression of Ppbec1, Ppxero2 and Pptha1

**Figure 2:**
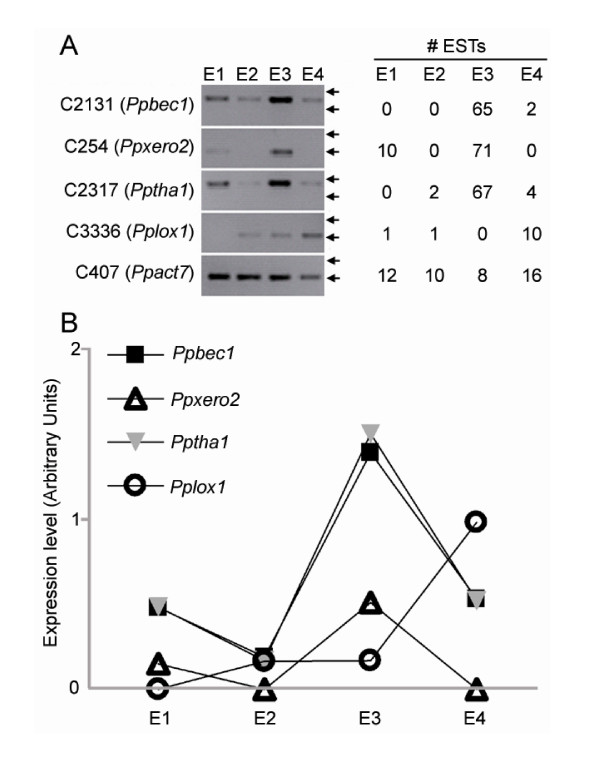
**Evaluation of the accuracy of the predicted expression patterns of selected genes by RT-PCR**. (A) RT-PCR analysis of RNA expression of three cold-induced genes: *Ppbec1*, *Ppxero2*, and *Pptha1 *under different post-harvest conditions. These post-harvest conditions include: fruits processed in a packing plant (E1: non-ripe; no long term cold storage); packing followed by a shelf-life at 20°C for 2-6 days (E2: Ripe; no long term cold storage; juicy fruits); packing followed by cold storage at 4°C for 21 days (E3: non-ripe; long term cold storage) and packing followed by cold storage at 4°C for 21 days and shelf-life at 20°C for 2-6 days (E4: Ripe; long term cold storage; woolly fruits). The expression level of *Pplox1 *was analyzed as a control for genes that do not express preferentially in cold stored fruits (E3). *Ppact7 *was analyzed as a control for genes that do not significantly change expression levels between the four post-harvest conditions analyzed. The two arrows associated with each gel represent 500 bp (upper) and 300 bp (lower). The number of ESTs associated with each contig and library source is indicated. (B) Densitometry quantification of the expression level obtained by RT-PCR, the figure shows the bands intensities for each gene relative to *Ppact7 *intensity.

We evaluated the expression levels of three cold-induced candidate genes by RT-PCR: a basic endochitinase (C2131, *Ppbec1*), a dehydrin (C254, *Ppxero2*) and a thaumatin-like protein (C2317, *Pptha1*). These genes were chosen due to the high number of ESTs in cold-stored fruits (E3), as revealed by the digital expression analyses (Figure 2). The expression level of a contig similar to lipoxygenase (C3336, *Pplox1*) that does not express preferentially in cold stored fruits (E3) as well as the expression level of a contig (C407, *Ppact7*) that does not significantly change expression under the different post-harvest conditions, were analyzed (Figure 2). Interestingly, all five genes analyzed showed an expression pattern significantly similar to the ones predicted by the digital expression analyses (Figure 2). The genes *Ppbec1*, *Ppxero2 *and *Pptha1 *have an increased expression in cold-stored fruits, whereas the *Pplox1 *gene increased expression in woolly fruits rather than cold-stored fruits.

### Identification of conserved motifs in the promoters of cold-inducible genes Ppbec1, Ppxero2 and Pptha1

We cloned 826 bp, 1,348 bp and 1,559 bp fragments corresponding to the regions upstream of the translation start codons of *Ppbec1*, *Ppxero2 *and *Pptha1*, respectively. The sequences of these promoter regions as well as the cDNA of their corresponding genes are shown in the Additional Files 2, 3 and 4.

The high sequence identity between the *Ppxero2 *contig with the coding region of *Ppdhn1*[30] was also observed within the promoter sequences of these two genes. Only one nucleotide difference at position -469 was found, suggesting that *Ppxero2 *and *Ppdhn1 *may be the same gene (Additional File 3). However, the promoter isolated in this work is about 230 bp longer (at the 5' end) than the previously published promoter [30].

**Figure 3:**
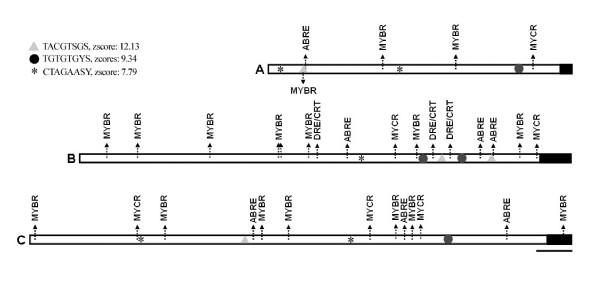
**Putative *cis*-regulatory elements identified in *Ppbec1*, *Ppxero2 *and *Pptha1 *promoter sequences**. Topologies of the *Ppbec1 *(A), *Ppxero2 *(B) and *Pptha1 *(C) promoters are shown. The promoters are draw proportionally (the bar correspond to 100 bp). Boxed regions: predicted 5' UTR region. Black arrow shows the position of different *cis*-regulatory elements related to low temperature responses: ABRE, DRE/CRT, MYBR and MYCR. The putative *cis*-regulatory elements identified by the motif prediction program YMF3.0 are shown as grey triangle, black circle and asterisk. The sequences, the symbol and the significance score (Zscore) of the motifs, are shown in the upper left corner. The degenerate bases allowed in the motifs are S (C or G) and Y (C or T). Note: in order to ensure at the legibility of the figure, not all cis-elements are marked in (B) and (C). However, the complete sequences of these promoters are available in Additional Files 3 and 4.

Cis-element regulatory motifs related to cold gene expression regulation such as ABRE [13], MYCR [31,32], MYBR [31,33] and DRE/CRT [34] were identified in all three promoters of these cold-inducible genes (Figure 3). In addition, three statistically significant predicted motifs were present in the promoters of these cold-inducible genes (TACGTSGS, TGTGTGYS and CTAGAASY (Figure 3). These motifs were not found in the *Pplox1 *promoter identified in this work (Additional File 5).

### Cold-induced Ppbec1 and Ppxero2 promoters in transiently transformed peach fruits and stably transformed Arabidopsis

**Figure 4:**
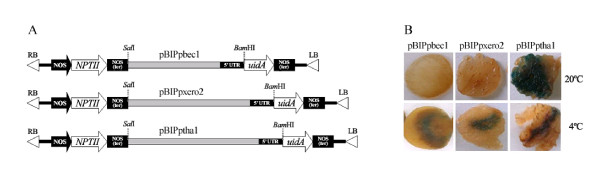
**Cold-inducible peach *Ppbec1 and Ppxero2 *promoters in transiently transformed peach fruits**. (A) Structure of the binary vector constructs used for functional analysis of the *Ppbec1*, *Ppxero2 *and *Pptha1 *promoter-*uidA *fusions. LB and RB: left and right T-DNA border. (B) Histochemical GUS staining of fruit slices from agro-infiltrated peaches stored at 20°C for 5 days post-inoculation or 4°C for 10 days. These images correspond to the transient transformation of *O'Henry *variety fruits. However, similar results were seen in all varieties assayed (data not shown).

Transient transformation assays of peach fruits revealed that all three cloned promoters (pBIPpbec1, pBIPxero2 and pBIPptha1) were able to activate GUS (*uidA*) expression (Figure 4). However, only the pBIPpbec1 and pBIPxero2 promoter constructs showed cold-inducible increases in GUS activity (Figure 4). The pBIPtha1 construct was expressed at both 20°C and 4°C. Comparable results were seen in fruits from three different peach varieties (data not shown).

**Figure 5:**
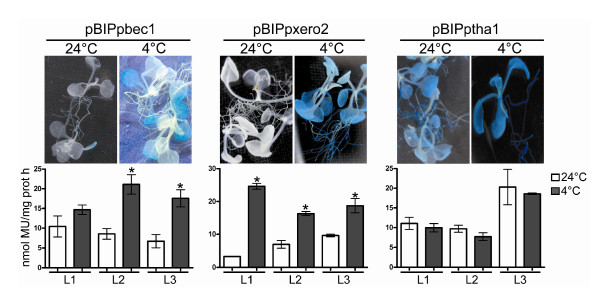
**Conserved heterologous regulation of the cold-inducible peach *Ppbec1 *and *Ppxero2 *promoters in transgenic Arabidopsis plants**. The upper panel shows histochemical GUS staining of representative transgenic Arabidopsis lines carrying the Ppbec1 promoter-uidA fusion, Ppxero2 promoter-uidA fusion and Pptha1 promoter-uidA fusion. The lower panel shows the results of fluorometric GUS-assays of three independent Arabidopsis transgenic lines (L1, L2 and L3) containing the Ppbec1 promoter-uidA fusion, Ppxero2 promoter-uidA fusion or Pptha1 promoter-uidA fusion. Homozygous T3 plants were grown for 14 days in MS plates with 0.8% agar at 24°C (white bars) and then transfer to 4°C for 7 days (blacks bars). The asterisk above each bar represents those samples that have a statistically significant increase in GUS activity in the cold treated plants when compared to the untreated plants. Bars represent the mean ± standard deviation, n = 5. t-student * p < 0.01.

Similar results were seen when these promoter-GUS constructs were analyzed in stably transformed Arabidopsis. All three constructs were able to activate GUS expression, but only the *Ppbec1 *and *Ppxero2 *promoters (pBIPpbec1 and pBIPxero2, respectively) induced expression in response to cold (Figure 5). As observed with the fruit transient transformation assays, the *Pptha1 *promoter (pBIPtha1) expressed GUS under all conditions analyzed.

## Discussion and Conclusion

Digital expression analyses of EST datasets have permitted us to identify a large diversity of cold-inducible genes in peach fruits, three of which were chosen for further analyses (*Ppbec1*, *Ppxero2 *y *Pptha1*). Both digital expression analyses and RT-PCR suggest that the *Ppbec1*, *Ppxero2 *and *Pptha1 *are cold-inducible genes. The promoters of these cold-inducible genes were isolated and characterized using both transient transformation assays in peach fruits and stable transformation in Arabidopsis. These analyses have revealed that the isolated *Ppbec1 *and *Ppxero2 *promoters are cold-inducible promoters, whereas the isolated *Pptha1 *promoter was not cold-inducible. These results, therefore, demonstrate that the isolated *Ppbec1 *and *Ppxero2 *promoters are sufficient for cold-induced gene expression. Furthermore, these results suggest that there is a conserved heterologous cold-inducible regulation of these promoters in peach and Arabidopsis.

Plants respond to cold temperatures by modifying the transcription and translation levels of hundreds of genes [35,36]. These acute molecular changes are related to plant cell physiological and biochemical modifications (cold acclimation) that lead to stress tolerance and cold adaptation (a chronic response). In peach fruits, cold temperatures induce chilling injury, possibly due to global transcriptome changes [37]. With the exception of studies in the model organism *A. thaliana *[4] and work published recently [17,38], little is known about the peach global transcriptional response to cold. Using the Pearson correlation coefficient, we analyze the coordinated gene expression of 1,402 contigs. This analysis revealed 164 genes preferentially expressed in peach fruits, of which digital expression analyses [18] revealed 45 of these genes (27%) with statistically significant cold-induction. A large proportion of the contigs preferentially expressed at 4°C (around 74% of the total) do not exhibited significant sequence homology (e-value < e^-10^) with the rest of the analyzed contigs (Table 2). This result could suggest that these contigs represent genes with non-redundant functions that will have a special importance during the exposure of the fruits to low temperatures.

Among the highly expressed genes in cold stored fruits, we found genes related to stress response in plants, including three dehydrins (C30, C254 and C304), three chitinases (C910, C2131 and C2441), four thaumatin-like proteins (C1708, C2177, C2317 and C2147), and polygalacturonase inhibiting protein (C2988), similar to what was reported by Ogundiwin et al [38]. Dehydrins are hydrophilic proteins that belong to the subgroup D-11 of the LEA ("late-embryogenesis-abundant") proteins [39]. There is some evidence that suggests that dehydrins protect macromolecules such as membranes and proteins against the damages associated with water deficiency [40-42]. In peach, these genes are induced during cold acclimation and in cold-stored fruits [30,38]. It has been observed that pathogenesis-related (PR) proteins such as chitinases and thaumatins are accumulated in the apoplastic space in winter rye during cold acclimation. These proteins also may have antifreeze properties that will protect the integrity of the plant cell avoiding the formation of ice [43,44]. It has also been observed that these types of proteins retain their enzymatic activity under low temperatures, and may form part of a general response mechanism associated with unfavorable conditions, by providing protection from opportunist pathogen attack whilst the plant is in a weakened state [45-47]. A similar role is shared by polygalacturonase inhibiting proteins in different plants models [48,49].

We also found some genes related to protein folding and degradation, such as heat shock proteins, BiP-1 and DJ-1 family proteins (Table 2). These processes are very active when plants face low temperatures, chemical and oxidative stress. These proteins participate in the prevention and repair of damage produced by cold, through the stabilization of protein structure and the degradation of proteins that are not folded correctly [50,51].

In this work we were interested in isolating and functionally characterizing promoters of cold-inducible peach genes. To date, only a few inducible promoters have been identified in crop plants. The *Pptha1*, *Ppbec1 *and *Ppxero2 *genes were chosen for promoter cloning and characterization based on the up-regulation that these genes showed in the *in silico *analysis and RT-PCR. The promoter sequences of these genes contain several *cis*-regulatory elements such as DRE/CRT, ABRE, MYCR (MYC recognition site) and MYBR (MYB recognition site) [13,31-34] that are related to stress response, specifically to cold/dehydration. These cis-regulatory elements are conserved in several plant species [52]. The presence of these conserved motifs suggests that these promoters may respond to the cold. Using transient transformation in peach fruit we confirmed that the promoters isolated from *Ppbec1 *and *Ppxero2 *are induced during low temperature storage, but not at room temperature. On the other hand, the *Pptha1 *promoter is active under all the temperatures analyzed. This could indicate that the *Pptha1 *promoter sequence might not contain all the elements needed to regulate expression in a cold-inducible manner. Alternatively, the agro-infiltration technique may induce stress signals that will activate this promoter. However, this last possibility is not likely because the activation of the *Pptha1 *promoter at all analyzed temperatures is also seen in the stably transformed transgenic Arabidopsis plants. The promoters *Ppbec1 *and *Ppxero2*, however, are cold-induced both in Arabidopsis transgenic plants as well as transient expressing fruits, suggesting that the *Ppbec1 *and *Ppxero2 *promoters are cold-inducible peach promoters. The cold-inducibility of these promoters in *A. thaliana *also suggests that this model plant may be used to functionally analyze peach cold-induced genes as well as their corresponding cis-elements and trans-acting factors.

The identification of these fruit tree cold-inducible promoters as well as the conserved heterologous regulation of these promoters in peach and Arabidopsis, demonstrates that these two transformation assays may be used to molecularly define the cis-elements and trans-acting regulatory factors that are associated with cold-responsive genes. By better understanding the regulatory mechanisms associated with cold-responsive genes, we may better understand the molecular differences and similarities between cold acclimation and chilling injury as well as the role these processes play in fruit tree growth and fruit quality.

## Authors' contributions

AT: identified and cloned the promoters. AT, MS, LM and HS drafted the manuscript. AT and MS: performed the digital expression analysis. AM and AT: performed the construction of Arabidopsis transgenic plants as well as the transient assay. HS: conceived, supervised and participated in all the analysis. All authors read and approved the manuscript.

## Supplementary Material

Additional file 1 **Identification of fruit cold-induced contigs using correlated expression analysis of peach ESTs**. The data provided represents the co-expression analysis of differentially expressed genes. The contigs were clustered using the Pearson linear correlation coefficient. Click here for file

Additional file 2 **Sequence of the *Ppbec1 *promoter and open reading frame**. The data provided represents the sequences of the *Ppbec1 *promoter and open reading frame. Click here for file

Additional file 3 **Sequence of the *Ppxero2 *promoter and open reading frame**. The data provided represents the sequences of the *Ppxero2 *promoter and open reading frame. Click here for file

Additional file 4 **Sequence of the *Pptha1 *promoter and open reading frame**. The data provided represents the sequences of the *Pptha1 *promoter and open reading frame. Click here for file

Additional file 5 **Sequence of the *Pplox1 *promoter and open reading frame**. The data provided represents the sequences of the *Pplox1 *promoter and open reading frame. Click here for file
