# Supplementary material for: Isolation and functional characterization of cold-regulated promoters, by digitally identifying peach fruit cold-induced genes from a large EST dataset
Source: BMC Plant Biol. 2009 Sep 22;9:121. doi: 10.1186/1471-2229-9-121 (PMC2754992; doi:10.1186/1471-2229-9-121)
Supplement: Additional file 1 — Identification of fruit cold-induced contigs using correlated expression analysis of peach ESTs. The data provided represents the co-expression analysis of differentially expressed genes. The contigs were clustered using the Pearson linear correlation coefficient. [file 1471-2229-9-121-S1.DOC]

**
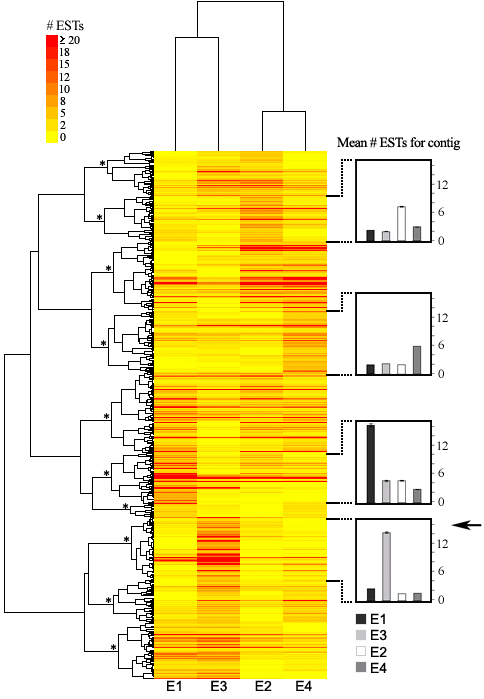
**

**Additional File 1: Identification of fruit cold-induced contigs using correlated expression analysis of peach ESTs.** 1,402 contigs with at least five ESTs were used to construct the clustered correlation map of the four postharvest cDNA libraries (Packing, non ripe: E1; Ripe Juicy: E2; Cold Storage: E3 and Ripe Woolly: E4). Color scale ranges from yellow for 0 ESTs to red for 20 or more ESTs. Expression profile of each contig is represented by a single row of colour boxes and the one of each library is represented by a single column. Ten major hierarchical clusters are indicated by an asterisk. Pearson correlation coefficients of all the gene expression profiles within ten major clusters are > 0.85. The mean number of ESTs constituting a contig in each cDNA library by the four hierarchical clusters of genes expressed preferentially in one cDNA library is shown as a histogram with standard error in the right. With an arrow are shown the 164 cold-induced contigs.
