# Supplementary material for: Isolation and functional characterization of cold-regulated promoters, by digitally identifying peach fruit cold-induced genes from a large EST dataset
Source: BMC Plant Biol. 2009 Sep 22;9:121. doi: 10.1186/1471-2229-9-121 (PMC2754992; doi:10.1186/1471-2229-9-121)
Supplement: Additional file 3 — Sequence of the Ppxero2 promoter and open reading frame. The data provided represents the sequences of the Ppxero2 promoter and open reading frame. [file 1471-2229-9-121-S3.DOC]

**
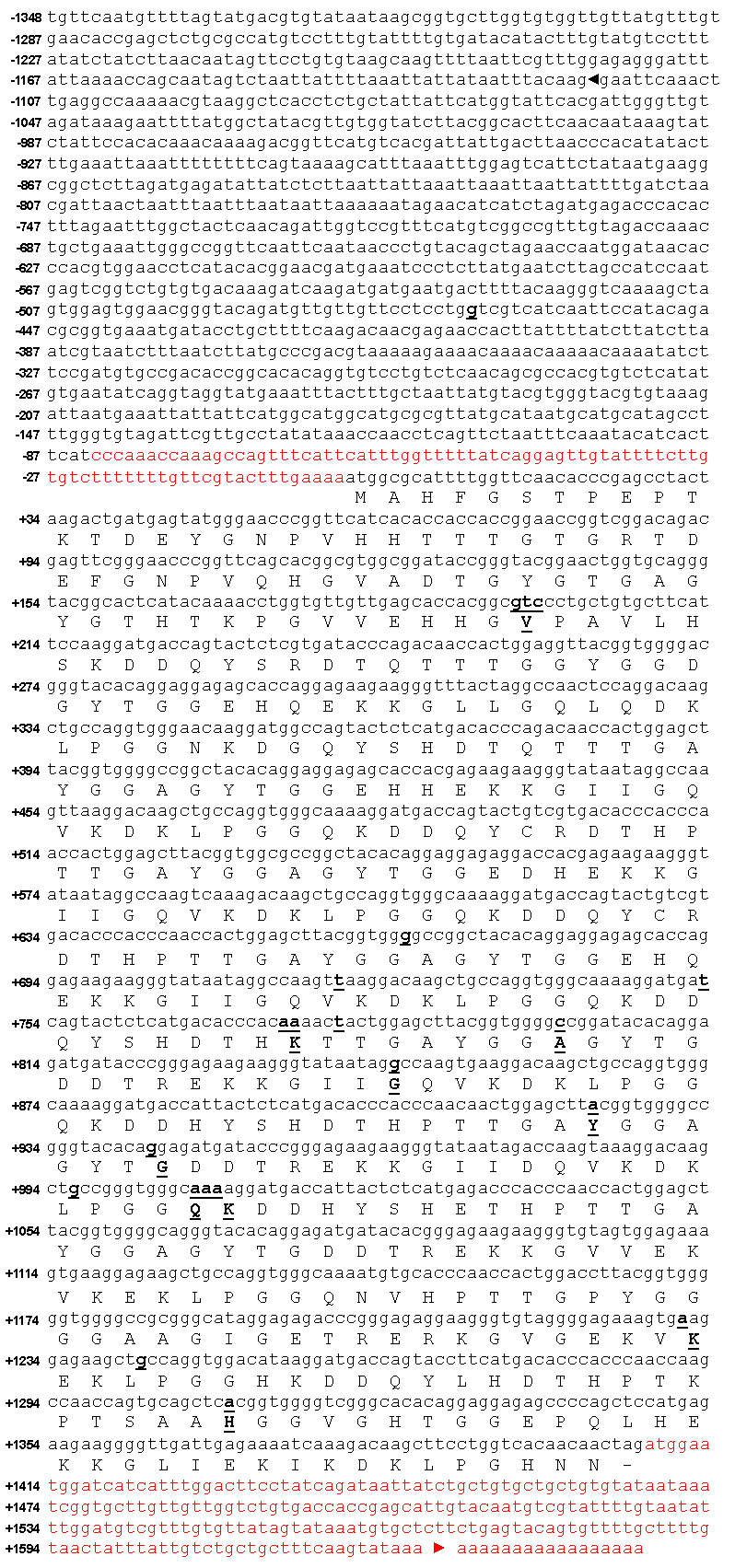
**

**Additional File 3: Sequence of the *Ppxero2* promoter and open reading frame**. The promoter sequence was fused virtually to the coding sequence of *Ppxero2* (C254). The sequences in red are the predicted unstranslated regions. The predicted protein sequence also is included. The sequences in dark and underlined (aminoacidic and nucleotidic) are different between *Ppxero2* and *Ppdhn1* (GenBank accession number: AAC49658). The published sequence of the promoter of *Ppdhn1* (GenBank accession number: AY819769) finish in the dark arrow, upstream from this arrow are the sequence isolated in this work.
