# Supplementary material for: Isolation and functional characterization of cold-regulated promoters, by digitally identifying peach fruit cold-induced genes from a large EST dataset
Source: BMC Plant Biol. 2009 Sep 22;9:121. doi: 10.1186/1471-2229-9-121 (PMC2754992; doi:10.1186/1471-2229-9-121)
Supplement: Additional file 4 — Sequence of the Pptha1 promoter and open reading frame. The data provided represents the sequences of the Pptha1 promoter and open reading frame. [file 1471-2229-9-121-S4.DOC]

**
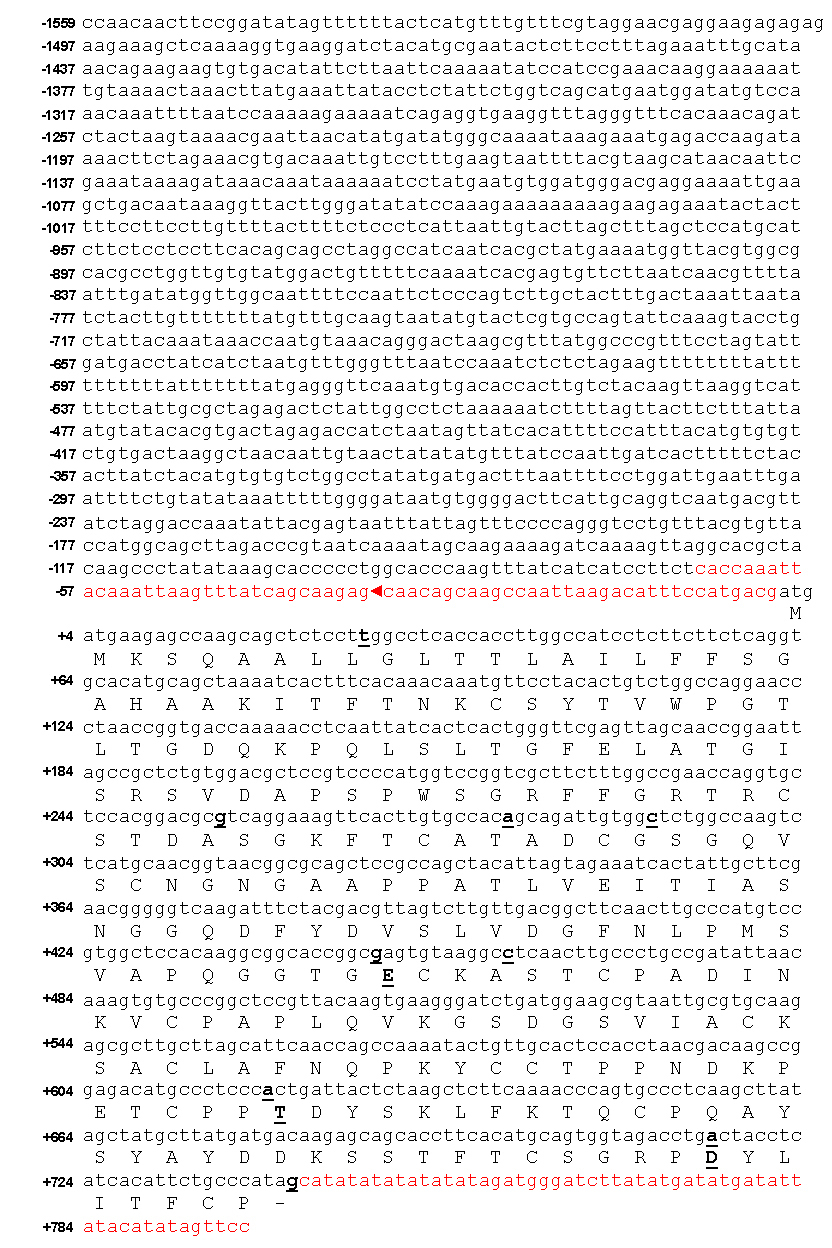
**

**Additional File 4: Sequence of the *Pptha1* promoter and open reading frame**. The promoter sequence was fused virtually to the coding sequence of *Pptha1* (C2317). The sequences in red are the predicted untranslated regions. The predicted protein sequence also is included. The sequences in dark and underlined (aminoacidic and nucleotidic) are different between *Pptha1* and the *Prunus persica* published thaumatin-like protein sequence (GenBank accession number: P83332). The published sequence of the thaumatin cDNA finished in the red arrow.
