# Supplementary material for: Isolation and functional characterization of cold-regulated promoters, by digitally identifying peach fruit cold-induced genes from a large EST dataset
Source: BMC Plant Biol. 2009 Sep 22;9:121. doi: 10.1186/1471-2229-9-121 (PMC2754992; doi:10.1186/1471-2229-9-121)
Supplement: Additional file 5 — Sequence of the Pplox1 promoter and open reading frame. The data provided represents the sequences of the Pplox1 promoter and open reading frame. [file 1471-2229-9-121-S5.DOC]

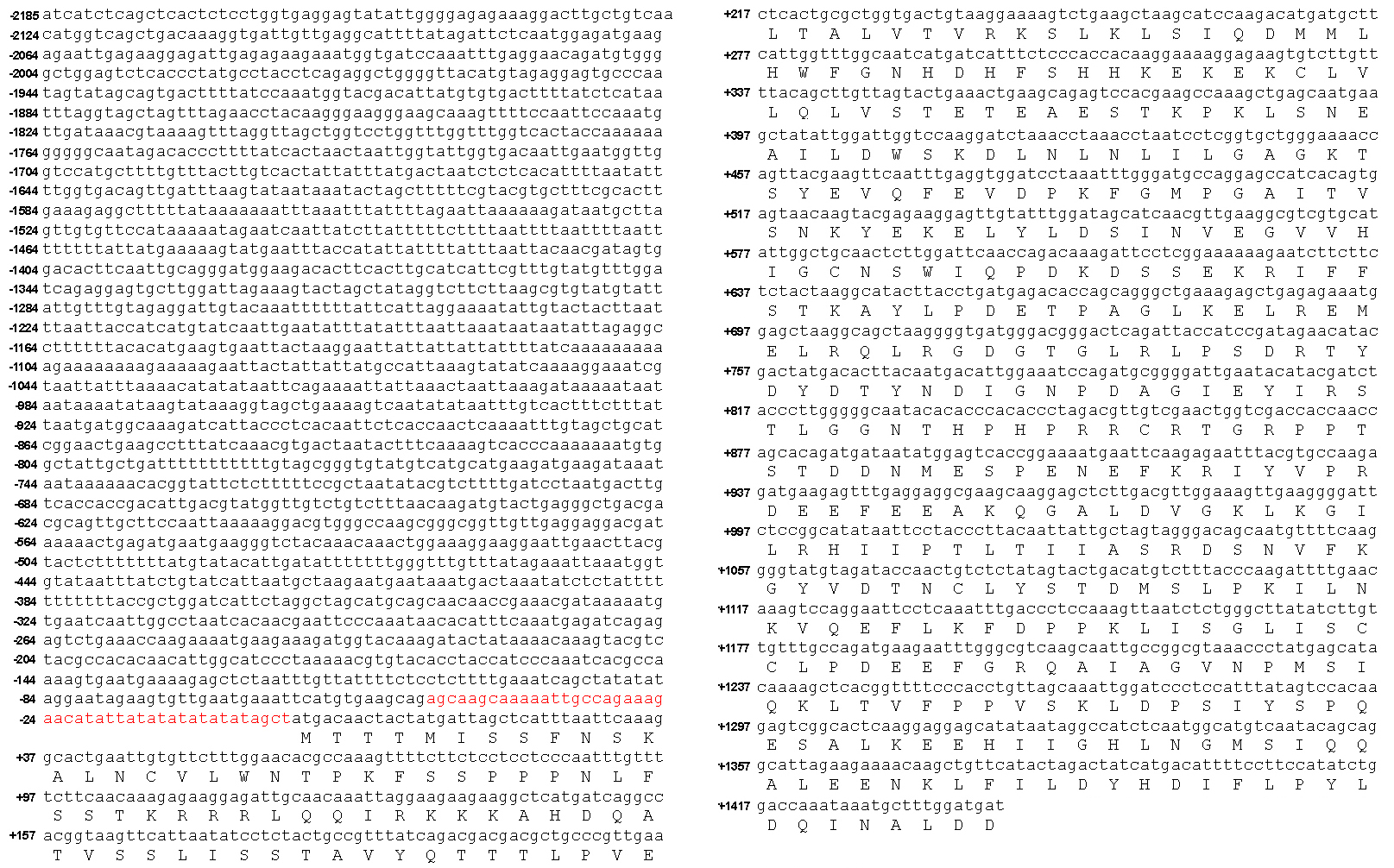


**Additional File 5: Sequence of the *Pplox1* promoter and open reading frame**. The promoter sequence was fused virtually to the coding sequence of *Pplox1* (C3336). The sequence in red is the predicted untranslated region. The predicted protein sequence also is included.
